# Composite free fibula flap and bone allograft for calcaneus reconstruction: A rare case

**DOI:** 10.1016/j.jpra.2025.03.004

**Published:** 2025-03-08

**Authors:** Gonçalo Tomé, Dmitry Shelepenko, José M. Azevedo, Inês Catalão, Carla Diogo

**Affiliations:** Plastic and Reconstructive Surgery and Burns Unit, Coimbra Local Health Unit, Coimbra, Portugal

**Keywords:** Microsurgery, Calcaneus, Composite, Fibula flap, Bone allograft

## Abstract

Calcaneus sarcomas are extremely rare, but often nefarious. Its weight-bearing function makes reconstruction after calcanectomy exceptionally difficult, demanding a solid, stable and well-padded substrate. We report a 69 years-old female with a rare small-round-cell sarcoma in her left ankle, submitted to total calcanectomy and reconstruction with a composite calcaneus allograft and free fibula flap. After 36 months, the bone healed evenly with no limitations in her daily life. This advantageous combination of vascularized bone with allograft, allowed biological integration with the strengthful external construct, representing one of the best limb-sparing options for the calcaneus.

## Introduction

Calcaneus bone and soft tissue sarcomas are remarkably rare, representing <1 % of these tumors.^1^ Foot compact structure and reduced compartmentalization dictates early adjacent tissue tumor involvement, often requiring total calcanectomy.^2^

Calcaneus reconstruction is exceptionally challenging, due to its weight-bearing role and inputted stress. Function restitution and durability are primary goals considering the patient's quality of life. Different reconstructive techniques include prothesis, bone allografts or vascularized bone transfers.^2^^,^^3^ Associating vascularized fibula with bone allograft combines the advantages of vascular bone healing integration with external compact bone, making it an utterly interesting option for significant weight-bearing defects.^4^

We intended to describe a rare case of a small-round-cell sarcoma submitted to total calcanectomy and reconstruction with combined free fibula flap and bone allograft, exploring its advantageous reconstructive potential and reviewing the literature on its use for the calcaneus.

## Case report

We present a 69 years-old female patient with a small-round-cell sarcoma in her left ankle, involving both soft tissue and calcaneus bone. The 5-month rapidly increasing tumorous bump in her ankle, caused pain and difficulty walking. She had type II diabetes and no other comorbidity. Preoperative study included tumor biopsy, computed tomography (CT) scan and magnetic resonance imaging, and angiography for vascular integrity evaluation.

**Figure 1:**
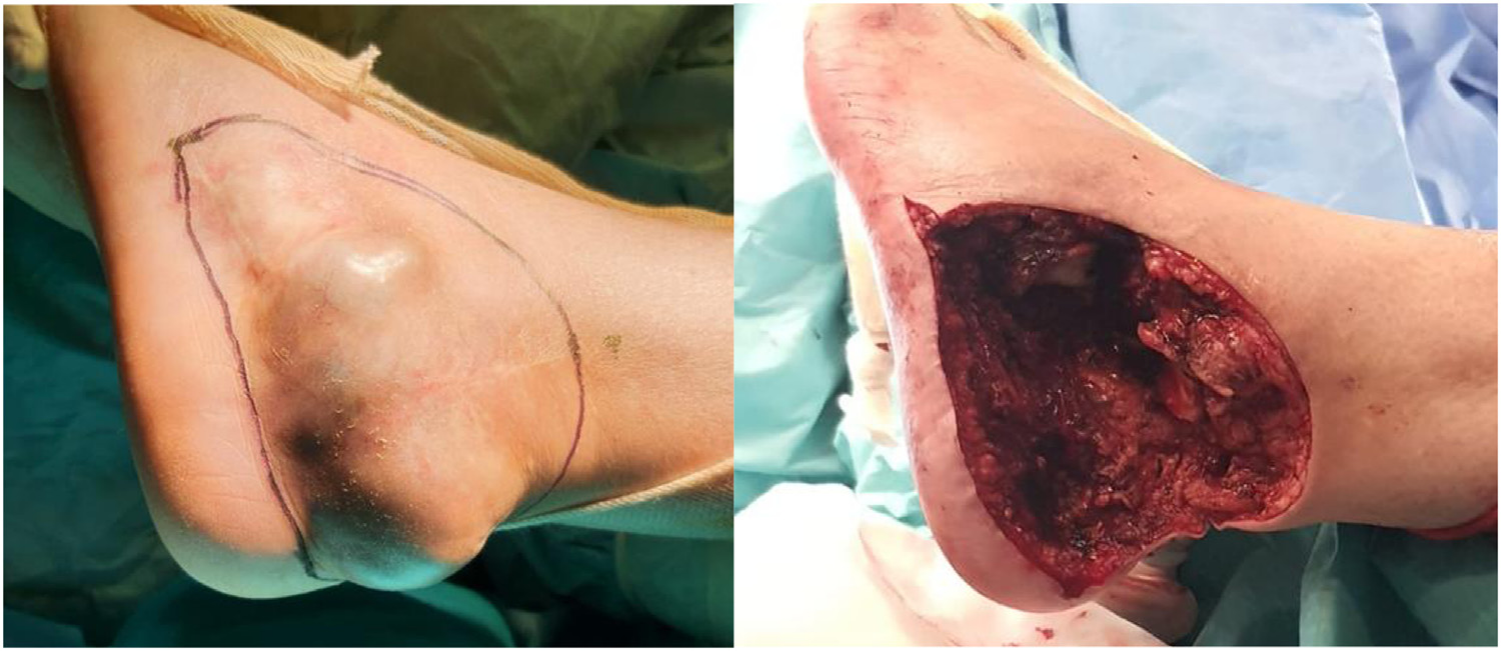
Ankle sarcoma (left) and defect after resection (right).

A double-team approach was used for ablation and reconstruction. Tumor wide resection with total calcanectomy through a lateral approach resulted in a 11 cm length defect (Figure 1). Hand-held doppler identified skin paddle perforators preoperatively. A thigh tourniquet was inflated, and the fibula flap harvested from her contralateral leg, leaving 6 cm of intact bone on both extremities.

**Figure 2:**
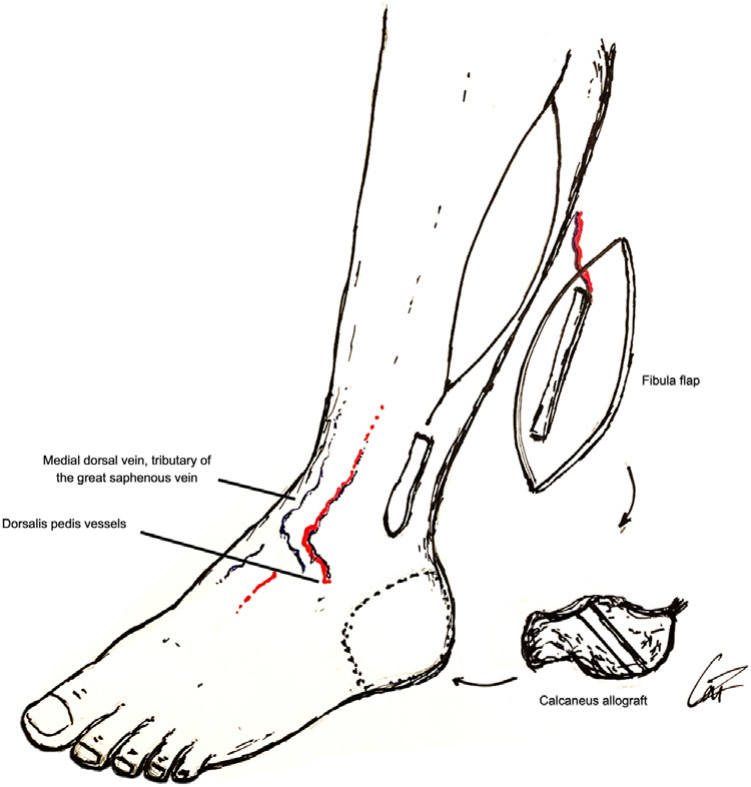
Schematic representation of the combined reconstruction.

**Figure 3:**
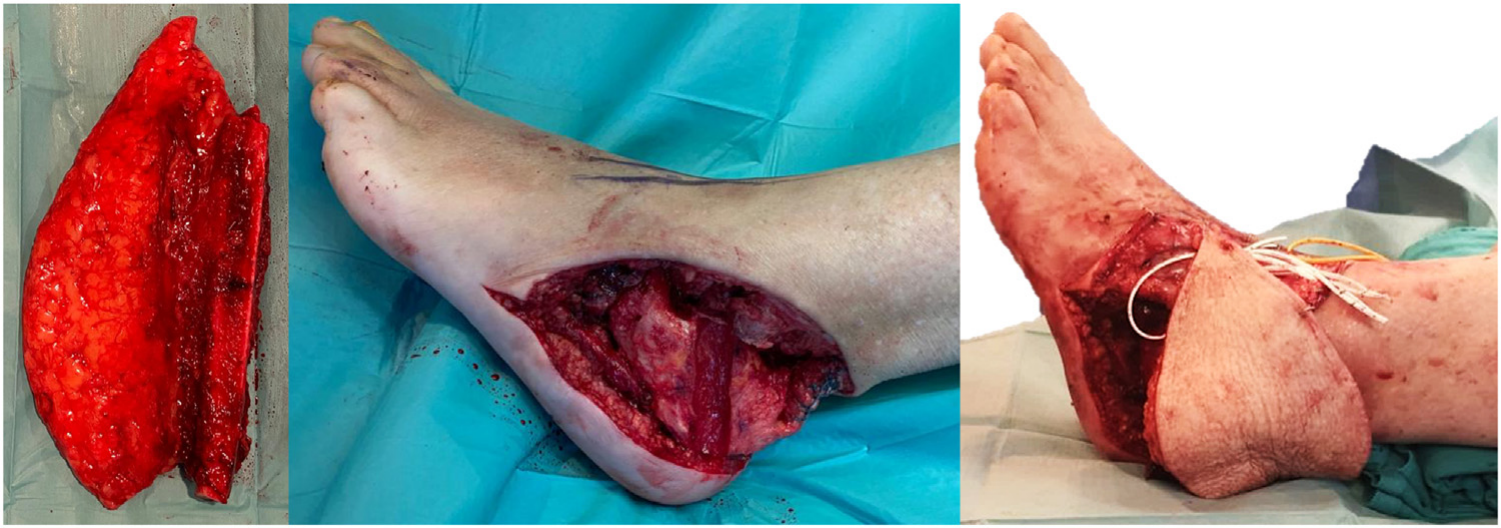
Fibula flap (left), trimmed calcaneus allograft placed in the tumor resection defect (middle) and fibula flap inset and encased in the allograft (right).

A similar size calcaneus allograft was prepared to have the fibula encased laterally on its body and shaped to match the recipient site, using a high-speed burr. Talus, calcaneus allograft and cuboid articular interface were debrided for arthrodesis. The trimmed free osteoseptocutaneous single barrel fibula flap was 6 cm long and its skin paddle measured 18 × 11 cm. Three cannulated screws allowed allograft fixation to the fibula, talus and cuboid bones. The Achilles tendon was sutured. Dorsalis pedis artery, one comitant vein and a medial dorsal vein of the foot were end-to-end microanastomosed with hand suture to the flap's pedicle (Figure 2, Figure 3). Donor site was closed with split thickness skin graft from the ipsilateral thigh.

After 14 h, the skin paddle developed signs of venous congestion and immediate revision surgery confirmed thrombosis in both veins but patency of the artery. The skin edges and subcutaneous fat were actively bleeding congestive blood, the artery was pulsating along its course and the milking test confirmed arterial microanastomosis patency. Both veins were cut proximally to the microanastomosis and vein end clots were removed. Veins started oozing more notoriously. Then, venous microanastomosis were redone with hand to the small saphenous and a different dorsal vein of the foot, followed by 5000 IU of unfractionated heparin administered via an intravenous catheter. Venous congestion was successfully reversed. No skin paddle necrosis was developed and there was no need for any debridement. Splints kept the donor leg immobilized for 2 wk and reconstructed leg for 2 months. Afterwards, she started physical rehabilitation with progressive ankle movement.

**Figure 4:**
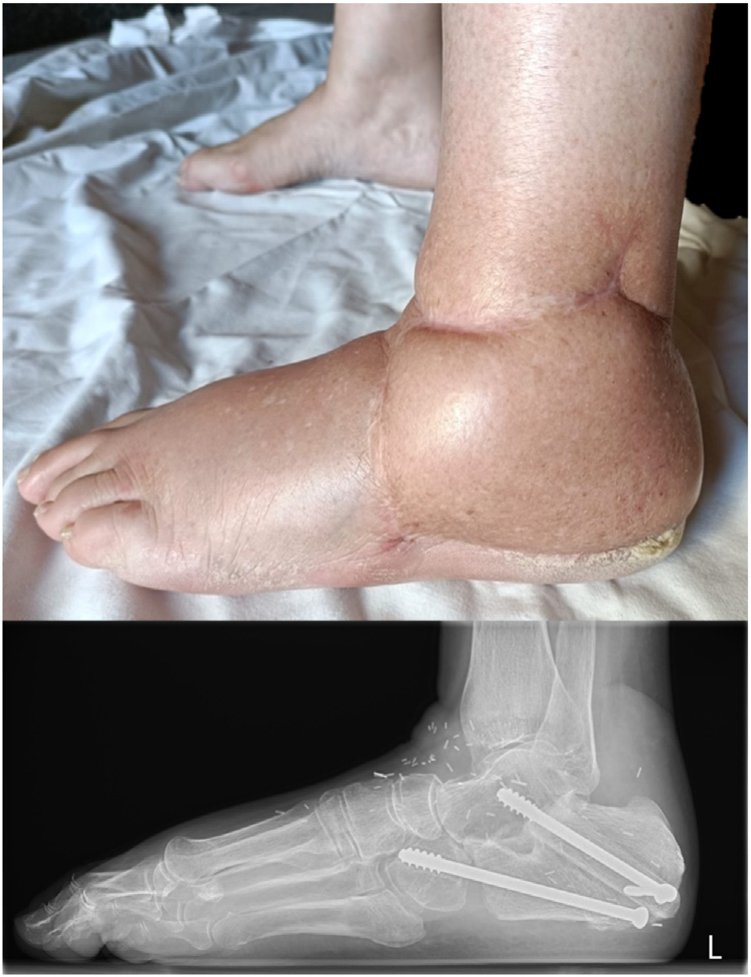
Thirty-six moths of follow up: flap contour (up) and radiography evidencing the fibula bone encased on the allograft, the 3-screw fixation and adequate bone healing (down).

Postoperative evaluations were conducted at every 2–3 months during the first year and every 4–6 months afterwards. Clinical, radiographic, CT scan and positron emission tomography scan evaluations were done to exclude recurrency and evaluate flap integrity. At 3 months, she started partial weight-bearing, at 6 months bone healing was achieved, and at 10 months progressed to full-bearing. In 36 months of follow-up, it remained well vascularized, healed evenly, well integrated in the allograft (Figure 4). In her last observation, she only mentioned occasional mild pain that did not limit her everyday activities. The walking perimeter and gait were not affected. No other complications, complaints or significant limitations were observed. Her Visual Analogue Scale (VAS) pain score was 3 and the Musculoskeletal Tumor Society (MSTS) score was 90 %.

The case was operated and followed by the senior author (CD).

## Discussion

The ideal reconstruction option should provide the best functional and most durable result as possible, with appropriate stability, heel pad, and aesthetic contour, particularly for the weight-bearing calcaneus.^3^^,^^5^

Custom-made prothesis and calcaneus or femoral head allografts are classic options when soft tissue coverage is possible, and adjuvant therapy not planed. Persisting pain, infection and collapse are possible consequences.^3^ Resection arthroplasty is a simple, low-cost, but sparsely used alternative, in which leg length discrepancy should be expected.^3^

Bone flaps have intrinsic advantages related with its vascularized nature remodeling potential, resulting in improved stability, healing, strength and durability.^2^, ^3^, ^4^, ^5^ Fibula flap is a paradigmatic option for long bones intercalary defects. Calcaneus reconstruction is no exception, since fibula is a straight cortical bone capable of hypertrophy in response to weight-bearing and stand this stress, also allowing for simultaneous soft-tissue coverage.^2^^,^^5^^,^^6^ It can be used either single or double barreled, and pedicled distally, obviating microanastomosis, or as a free flap which allows for double-team approach and save operative time.^2^^,^^5^^,^^6^ Deep circumflex iliac artery (DCIA) osteocutaneous flap is a similarly good option that provides solid massive iliac bone coupled with a sufficiently generous soft-tissue pad for heel support.^7^^,^^8^

Associating vascularized fibula with bone allograft combines the strength and stability of an external bone allograft with the remodeling and integration capability of a vascularized bone. Fibula can be easily encased in the allograft, share the weight-bearing stress, develop hypertrophy and protect the allograft from collapse during its remodeling. The allograft also allows Achilles tendon reconstruction.^6^^,^^9^^,^^10^

We reviewed the literature on this composite reconstruction for the calcaneus after tumor resection (Supplementary 1 and 2).^9^^,^^10^ Six cases were reported combining bone allografts with pedicled (*n* = 5) or free (*n* = 1) fibula flaps. The follow-up was long (>2 years) and no relapse, relevant complication, failure or amputation were observed, similar to our case. Notably, composite reconstruction MSTS score was globally high (87–97 %),^9^^,^^10^ like DCIA flap (90–97 %),^7^^,^^8^ and higher than fibula flap alone (76–90 %), probably related with their more compact, solid and better padded construction.

Bone flaps require a delay in weight-bearing until its integration, are technically demanding, and have a risk for failure. However, compared to single bone allografts or prosthesis, the vascularized bone, particularly when composite, tend to have more durable results with less long-term complications. They can also provide soft-tissue coverage and be used when adjuvant therapy is planned.^2^^,^^3^^,^^5^, ^6^, ^7^, ^8^, ^9^, ^10^

Overall aesthetic and functional results with composite reconstruction were excellent, with very little to no limitations in daily activities, and to the best of our knowledge, our case represents the oldest person reported successfully submitted to this reconstruction.^9^^,^^10^

Our case presented excellent functional and aesthetic result, comparable bone union and weight-bearing times, no limitations in daily activities or relevant complications over a long follow-up period, corroborating the feasibility and efficacy of composite allograft and fibula flap as one of the best limb-sparing options for the calcaneus.

## Ethical approval

The present study follows the International Ethics Guidelines of Declaration of Helsinki and Council for International Organizations of Medical Sciences and no Ethical approval was required by the Ethics Committee.

## Patient consent statement

The patient has given her informed consent and permission to use of her clinical information and photographic material pertaining the present study.

## Conflict of interest

No conflict of interest to declare.

## Appendix Supplementary materials

Image, application 1

Image, application 2
