# Supplementary material for: Composite free fibula flap and bone allograft for calcaneus reconstruction: A rare case
Source: JPRAS Open. 2025 Mar 8;44:133–8. doi: 10.1016/j.jpra.2025.03.004 (PMC11984531; doi:10.1016/j.jpra.2025.03.004)
Supplement: Supplementary file 1 [file mmc1.docx]

Supplementary files

Supplementary 1. Literature review flow diagram

**Identification of studies via databases**

Records removed *before screening*:

Duplicate records removed

(n = 21)

Records identified from*:

Pubmed (n = 50)

Embase (n = 59)

Cross-reference (n = 1)

**Identification**

Records screened

(n = 89)

Records excluded

(n = 60)

Reports without full text available

(n = 3)

Reports sought for retrieval

(n = 29)

**Screening**

Reports excluded:

German (n = 1)

Review or systematic review (n = 2)

No bone, non-vascularized or allograft bone (n = 6)

Not relatable (n = 6)

DCIA flap (n=4)

Other flaps than fibula (n=2)

Reports assessed for eligibility

(n = 26)

Studies included in review

(n = 5)

**Included**

Pubmed and Embase databases were assessed, using the terms “bone”, “vascularized”, “calcaneus” and “tumor” for studies published until 31^st^ November 2024, yielding 110 articles consisting of case reports and case series. The studies were independently evaluated by the authors GT and DS, following PRISMA guidelines. Title and abstract were screened and articles without any detailed case, not including bone flaps for calcaneus, written in languages other than English or Portuguese and not relatable to our study were excluded. Subsequently, papers were retrieved to be assessed for eligibility, and 5 studies using fibula flap were included.
