# Supplementary material for: Composite free fibula flap and bone allograft for calcaneus reconstruction: A rare case
Source: JPRAS Open. 2025 Mar 8;44:133–8. doi: 10.1016/j.jpra.2025.03.004 (PMC11984531; doi:10.1016/j.jpra.2025.03.004)
Supplement: Supplementary file 2 [file mmc2.docx]

Supplementary files

Supplementary 2. Literature review of vascularized bone flaps for the calcaneus

| **First author and year** | **N** | **Diagnosis** | **Age (mean, range; years)** | **Reconstruction** | **Bone healing (mean, range; months)** | **Full weight- bearing (months)** | **Follow-up (mean, range; years or months)** | **Complications** | **MSTS scores (mean, range; %)** | **Result** |
| --- | --- | --- | --- | --- | --- | --- | --- | --- | --- | --- |
| Li J, 2010^5^ | 5 | Chondrosarcoma (n=2) Ewing sarcoma (n=2) Osteosarcoma (n=1) | 23 (16-43) | Pedicled flap Distally pedicled osteocutaneous folded (double barrel) fibula flap | 6.2 (5-8) | 8.6 (7-11) | 32-76 mo | Hematoma (n=1) and skin margin necrosis (n=1) | 83.2 (76-90) | All fibular flaps survived, and arthrodesis healed successfully. Fibula hypertrophies were seen in 3 patients. All but one patient had no limitation in daily activities and run and jump |
| Li J, 2012^9^ | 4 | Chondrosarcoma (n=1) Fibrosarcoma (n=1) Osteoblastoma (n=1) Giant-cell tumor (n=1) | 32.1 (27–36) | Composite Allograft (calcaneus or trimmed distal femur) and osteocutaneous free (n=1) and pedicled fibula flap (n=3) | 5.9 (4.5-7) | 7 (6.5-8) | 24.5 (13-37) mo | Skin flap necrosis (n=1) Infection (n=1) | 91.7 (87-97) | Pain completely absent, all patients could walk more than half an hour and could go upstairs without any support. No problem in single leg stance |
| Li J, 2014^2^ | 5 | Chondrosarcoma, osteosarcoma, Ewing sarcoma | NA | Pedicled flap Distally pedicled osteocutaneous folded (double barrel) fibula flap | NA | 8.6 | 42.3 mo | Hematoma (n=1) Skin margin necrosis (n=1) Temporary pain (n=1) Decreased range of motion (n=1) | 83.2 | Pain completely absent in all the patients. No special shoes were needed. No evident limp or limitation of daily activities in 4 patients, mild limp in 1 patient. |
| Ruiz-Moya A, 2019^6^ | 1 | Ewing sarcoma | 6 | Pedicled flap Distally pedicled osteocutaneous (single barrel) fibula flap | 6 | 10 | 68 mo | Wound dehiscence | NA | Full weight bearing and capable of doing sports, walk and run. Limb discrepancy of 2 cm |
| Hamrouni N, 2023^10^ | 2 | Ewing sarcoma | 5-16 | Composite Allograft (calcaneus or femoral head) and distally pedicled osteocutaneous fibula flap | 3,5-6 | 8 | 7-10 y | Delayed wound healing (n=1) | NA | Near-normal ambulance |

Legend: DCIA: Deep circumflex iliac artery; mo: months; MSTS: Musculoskeletal Tumor Society Score; NA: not available; y: years.
